# Minimizing near-infrared autofluorescence in preclinical imaging with diet and wavelength selection

**DOI:** 10.1117/1.JBO.28.9.094805

**Published:** 2023-04-05

**Authors:** Yidan Sun, Xingjian Zhong, Allison M. Dennis

**Affiliations:** a Northeastern University, Department of Chemical Engineering, Boston, Massachusetts, United States; b Boston University, Department of Biomedical Engineering, Boston, Massachusetts, United States; c Boston University, Department of Materials Science and Engineering, Boston, Massachusetts, United States

**Keywords:** preclinical imaging, near-infrared, short-wave infrared, fluorescence, autofluorescence, NIR-II, deep tissue imaging

## Abstract

**Significance:**

Preclinical fluorescence imaging with NIR-I (700 to 900 nm) illumination and short-wave infrared or NIR-II (1000 to 1700 nm) emission increases tissue penetration depth and improves resolution through decreased scattering. Background autofluorescence decreases signal-to-background ratios (SBR) in fluorescence imaging; maximizing SBR will further improve the impact of deep tissue imaging.

**Aim:**

The impact of rodent diet, illumination wavelength, and emission range on the background fluorescence and contrast agent SBR were determined to assist with the experimental design of future imaging studies.

**Approach:**

Following illumination with 670, 760, or 808 nm, autofluorescence in the NIR-I (<975  nm), NIR-II (>1000  nm), and NIR-II LP (>1250  nm) regions was assessed in mice fed chow or a purified diet using an IR VIVO preclinical imager (Photon, Etc.). Comparison of the SBR of liver-localized indocyanine green in the various imaging conditions indicated when gut autofluorescence was a problematic confounder.

**Results:**

Mice fed chow exhibit high levels of background autofluorescence in the gastrointestinal tract and, to a lesser extent, skin when illuminated with 670 nm light for NIR-I imaging (700 to 975 nm), interfering with the identification of fluorescently labeled tissue. Background autofluorescence was reduced by more than two orders of magnitude by any of the following changes: (1) purified diet; (2) excitation with 760 or 808 nm illumination; or (3) emission in the NIR-II (1000 to 1600 or 1250 to 1600 nm). Although the SBR was generally sufficient for feature identification except when imaging of chow-fed mice with 670 nm excitation and NIR-I emission, switching to a purified diet, using longer excitation wavelengths, or using longer emission wavelengths improved SBR significantly.

**Conclusions:**

Systematic comparison of imaging conditions and diet highlights the reduction in autofluorescence and increase in SBR enabled by intentional choices in the experimental parameters including diet, excitation wavelength, and emission wavelength range.

## 1 Introduction

*In vivo* fluorescence imaging is a widely adopted tool because it is noninvasive and uses low-cost equipment and nonionizing radiation (i.e., light).^1^^,^^2^ The modality offers high sensitivity, high framerate visualization of organs, tissues, cells, and molecules for applications such as disease diagnosis, image-guided surgery, and *in vivo* sensing.^3^^–^^5^ In the visible wavelength range, fluorescence imaging suffers from poor resolution and limited signal penetration depth caused by tissue absorbance, scattering, and autofluorescence. Shifting to a longer wavelength range, namely the near-infrared I (NIR-I) biological window (700 to 900 nm), reduces tissue scattering and light absorbance from key biological molecules, such as hemoglobin and oxygenated hemoglobin, to improve imaging depth.^6^^,^^7^ However, even in the NIR window, tissue autofluorescence can interfere with imaging outcomes. Challenges arise when high background tissue autofluorescence confounds the precise demarcation of the targeted region of interest by a contrast agent and reduces signal-to-background ratios (SBRs), ultimately leading to an impairment in imaging sensitivity.^8^ Methods to distinguish tissue autofluorescence from the contrast agent include signal separation using time-gated imaging or spectral unmixing.^9^^,^^10^ Another strategy is to expand the imaging window to even longer wavelength ranges, namely the NIR-II window (1000 to 1700 nm), also known as short-wave infrared (SWIR), where imaging also benefits from further reduced tissue scattering.^11^^–^^13^ These imaging advancements are enabled by recent developments in NIR-II-emitting fluorescent contrast agents and commercially available indium gallium arsenide (InGaAs) cameras for signal acquisition.^14^^–^^16^

A major contributor to tissue autofluorescence in NIR-I whole animal imaging is chlorophyll from the alfalfa component in regular mouse chow.^17^^–^^20^ Previous studies have shown that regular chow exhibits an emission feature around 660 nm attributed to chlorophyll with an excitation feature around 410 nm.^21^^–^^23^ Shifting the excitation to the deep red or NIR-I for NIR imaging results in an emission tail that extends into the NIR-I and NIR-II.^8^^,^^9^ Consistent with the ingestion of optically active molecules, autofluorescence is primarily observed from the digestive system in the abdomen, with less intense autofluorescence observed from skin.^18^ Previous studies mention reducing autofluorescence through experimental choices, such as changing diet or imaging, in the NIR-II; however, few studies systematically compare autofluorescence between NIR-I and NIR-II imaging or consider whether the purified diet is beneficial for NIR-II imaging.^11^^,^^24^^,^^25^ Diet, excitation wavelength, and emission wavelength range are all variables that can be chosen to optimize imaging experiments based on the study needs, available equipment, and contrast agent properties. With an understanding of how each of these experimental parameters impacts the background signal, astute choices can be made to enhance image contrast and SBR.

**Fig. 1:**
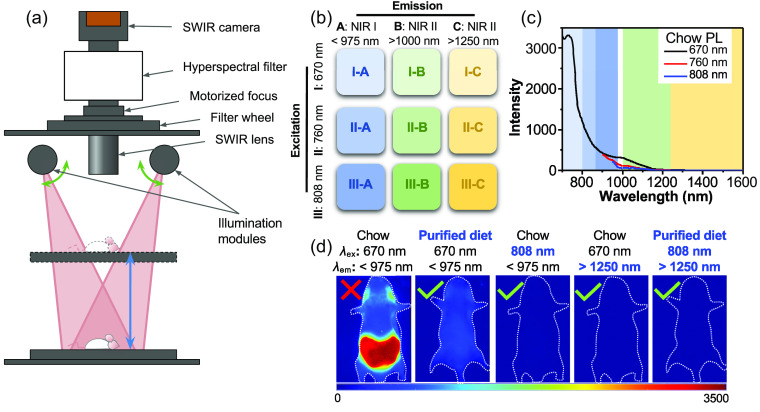
Summary of experimental setup and results. (a) Schematic of the key components of the IR VIVO, courtesy of Photon, Etc. Optics in the illumination modules ensure that the laser source is projected as rectangle-shaped homogenous illumination covering the field of view, the size of which varies depending on the position of the motorized z stage. Light from the mouse is collected through an SWIR lens, filtered through the filter wheel and/or a hyperspectral filter (HyperCube volume Bragg grating), and detected using a blue-extended InGaAs camera. (b) The excitation lasers and emission filters yield nine distinct imaging conditions by combining three excitation wavelengths (670, 760, and 808 nm) and three emission ranges (NIR-I, NIR-II, and NIR-II LP). The NIR-I emission ranges are 700 to 975 nm, 800 to 975 nm, and 875 to 975 nm for excitation with 670, 760, and 808 nm, respectively. The NIR-II is 1000 to 1630 nm, and the NIR-II LP covers 1250 to 1630 nm. (c) Photoluminescence (PL) spectra taken from hyperspectral images of a chow pellet using the three illumination sources. The background color in the graph correlates to the emission ranges used in the experiment. (d) Representative images showing how autofluorescence can be mitigated by changing one or more experimental parameters.

In this study, we examine autofluorescence in the NIR-I and NIR-II optical tissue imaging windows using multispectral and hyperspectral modes of an IR VIVO preclinical imager (Photon, Etc., Montreal, Canada; Fig. 1). We explore the difference in signal intensity using laser excitation sources at 670, 760, and 808 nm to examine the impact of excitation wavelength on autofluorescence across three emission ranges: NIR-I (<975  nm), NIR-II (>1000  nm), and NIR-II long pass (NIR-II LP, >1250  nm). Live and *ex vivo* organ imaging comparing mice fed regular mouse chow or a purified diet demonstrate the impact of diet on tissue autofluorescence. Hyperspectral imaging shows the red tail of autofluorescence, enabling the selection of filters to avoid it completely. Finally, imaging of the FDA-approved NIR dye indocyanine green (ICG)^26^^–^^28^ accumulated in the liver demonstrates the impact of diet, excitation wavelength, and emission wavelength range on image contrast.

## 2 Materials and Methods

### 2.1 Materials

The standard chow fed to mice was the 5P75 Prolab^®^ IsoPro^®^ RMH 3000 22% protein diet from Lab Diet (St. Louis, Missouri, United States). The “purified diet” was the OpenStandard Diet without dye D11112201N from Research Diets, Inc. (New Brunswick, New Jersey). ICG (≥95% purity) was purchased from Cayman Chemical Company (Ann Arbor, Michigan, United States), dissolved in DMSO (99.9%, Sigma-Aldrich), and diluted in sterile saline (Hospira, Inc.) for injection.

### 2.2 Animals

BALB/c nude mice (male and female) aged 3 to 4 weeks were purchased from Jackson Laboratory (Bar Harbor, Maine, United States). Mice were housed in the University Animal Science Center with standard diurnal lighting patterns, social housing, and *ad libitum* access to food and water. Mice were divided into two groups and fed different diets: standard chow or a purified diet that removes chlorophyll for at least one week prior to imaging. All procedures involving animals were reviewed and approved by the Institutional Animal Care and Use Committee of Boston University (IACUC Protocol No. PROTO201800466).

### 2.3 Preclinical Imaging

Mice were imaged with an IR VIVO preclinical imager (Photon Etc., Montreal, Canada) equipped with 670, 760, and 808 nm laser excitation sources, long-pass (LP) emission filters to eliminate scattered excitation light (700, 800, and 875 nm LP, respectively), and three emission filter options designated NIR-I (975 nm short-pass), NIR-II (1000 nm LP), and NIR-II LP (1250 nm LP) for multispectral imaging. The imager uses an InGaAs detector (ZephIR^™^ 1.7×, Photon Etc.) with a specified spectral range (QE>10%) of ∼500 to 1630 nm at the −80°C operation temperature. This study used a laser power density of $1\;\mathrm{mW}/{\mathrm{mm}}^{2}$ for all three excitation wavelengths and a lens setting of 50 mm f/5.0 with a 80×64  mm field of view. In each imaging sequence, a camera dark count background image was collected and subtracted from the image taken with excitation illumination, thereby facilitating time-matched background correction. Mice were imaged with an IR VIVO imager using 9 different excitation/emission combinations: 3 excitation wavelengths (670, 760, and 808 nm) × 3 emission ranges (NIR-I, NIR-II, and NIR-II LP). The NIR-I emission range varied depending on the excitation wavelength, as the LP filter used to exclude the excitation light changed to accommodate the excitation source. Specifically, the NIR-I emission range was 700 to 975 nm, 800 to 975 nm, and 875 to 975 nm for excitation with 670, 760, and 808 nm, respectively. The NIR-II emission included 1000 to 1630 nm, and the NIR-II LP range encompassed 1250 to 1630 nm.

For hyperspectral imaging, a volume Bragg grating (HyperCube, Photon, Etc.) was used to collect a series of images with a narrow emission range. Following dark background subtraction, wavelength registration, and image rectification in the PHySpec control and analysis software (Photon, Etc.), spectra of specific regions of interest (ROIs) were extracted from the image cubes. Images were visualized and analyzed in the PHySpec software and ImageJ (version 1.53t).

### 2.4 Imaging with Indocyanine Green

Mice fed chow or a purified diet were intravenously injected with a clinically relevant dose of ICG (0.5  mg/kg) and imaged 10 min post injection. Because of the low autofluorescence signal in the III-C filter with 808 nm excitation and >1250  nm emission, a rough ICG distribution area could be drawn according to the fluorescence pattern as indicated with the black solid line. Lower scattering at the longer wavelengths made the boundary clear, and it was easier to delineate the distribution of ICG and reveal the outline of the liver.

### 2.5 Statistical Analysis

Results were expressed as the mean ± standard deviation unless otherwise stated. The unpaired t-test was used for two-group comparisons of the autofluorescence images using GraphPad Prism 6 (GraphPad Software Inc., California, United States), and p-values<0.05 were considered statistically significant. Fluorescence SBRs were plotted and analyzed using JMP Pro v.15.0.0. Two-group comparisons were made using a t-test assuming unequal variances; three-group comparisons were made using Tukey–Kramer honestly significant difference (HSD) test.

## 3 Results and Discussion

**Fig. 2:**
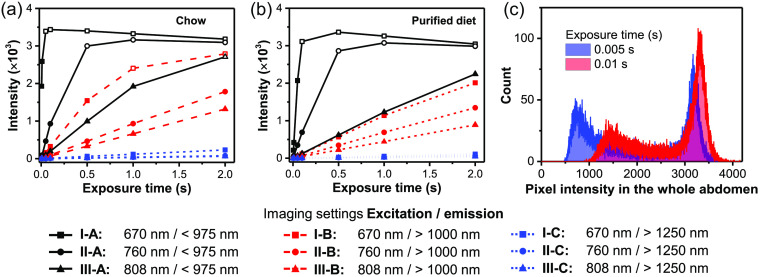
Time to image saturation under different imaging conditions. Average abdominal fluorescence intensity in mice fed (a) chow or (b) purified diet with different imaging exposure times (0.005, 0.01, 0.05, 0.1, 0.5, 1, 2 s). Excitation wavelength: 670 nm (I), 760 nm (II), and 808 (III). Acquisition filters: NIR I (<975  nm, A), NIR II (>1000  nm, B), and NIR II LP (>1250  nm, C). Open symbols indicate saturated data points. (c) Fluorescence saturation in the abdomen with 670 nm excitation and NIR-I emission (I-A). Comparison of pixel fluorescence intensity of the whole abdomen in mice fed chow with different exposure times. There is no cutoff intensity of the detector, but it shows a nonlinear response when the signal is higher than ∼3000.

To define the working range of the imager, we acquired a series of fluorescence images using each of the nine imaging settings with different exposure times and quantified the abdomen signal intensity (Fig. 2). Mice fed chow exhibited much brighter autofluorescence than those fed a purified diet under most imaging conditions; in fact, we observed detector saturation across a swath of pixels after only 0.05 s exposure when examining NIR-I emission with 670 nm excitation (I-A). This excitation/emission setting is the most similar to traditional NIR-I imaging such as with an *in vivo* imaging system (Perkin Elmer) and NIR dyes such as Cy7 or Alexa Fluor 750.^29^ For exposure times that avoided widespread detector saturation, the mean abdominal fluorescence intensities were proportional to the imaging exposure times. For most subsequent comparisons across imaging conditions, we chose the longest exposure time that avoided most detector saturation: 0.01 s.

Additionally, we observed autofluorescence in both female and male mice to assess if sex differences were likely to be a concern. Autofluorescence intensities in male and female mice showed little difference from each other in either chow group or purified-diet group aside from a shift in the location of genital autofluorescence consistent with the sex-dependent anatomy (data not shown). Following this comparison, we used mixed sex groups for subsequent assessments.

**Fig. 3:**
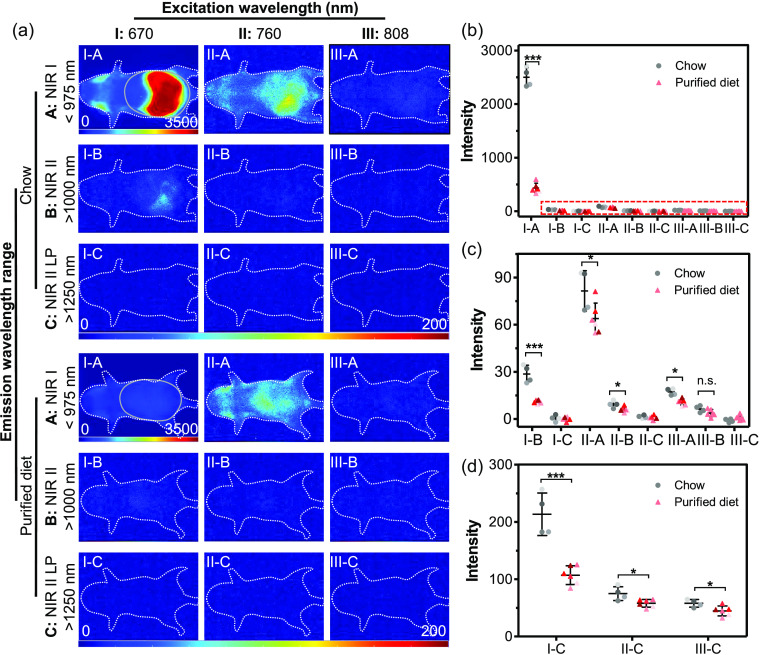
Impact of diet, excitation wavelength, and emission filter on mouse autofluorescence. (a) Representative images taken with 0.01 s exposure time. White dotted lines outline the position of the mice; gray solid ovals indicate the region used for quantitative analysis of abdominal autofluorescence. Excitation wavelength: 670 nm (I), 760 nm (II), and 808 nm (III). Acquisition filters: NIR-I (<975  nm, A), NIR-II (>1000  nm, B), and NIR-II LP (>1250  nm, C). Note that the intensity scale for the I-A images is from 0 to 3500 counts and all other images are on a scale of 0 to 200 counts. (b) Average abdominal autofluorescence pixel intensity with 0.01 s exposure time, as represented in (a). (c) Enlarged plot of data within the red dotted box. (d) Average abdominal autofluorescence intensity of mice imaged with NIR-II LP (>1250  nm, C) and long exposure time (2 s). Chow-fed group: n=4; purified diet-fed group: n=6. *p<0.05, **p<0.01, ***p<0.001.

When examining the average autofluorescence in the abdomen of mice fed different diets, the strongest signals in both groups appeared with 670 nm excitation and NIR-I emission (I-A), followed by imaging with 760 nm excitation and NIR-I emission (II-A); the fluorescence signal from other imaging conditions was notably weaker. In the I-A condition, average fluorescence intensity in chow-fed mice reached >5.6 times that in mice with the purified diet, with some pixels saturating the detector even at the shortest exposure time used (0.005 s) (Fig. 2). Compared to imaging with 670 nm excitation and NIR-I emission (I-A), changing to *either* longer excitation (760 nm or 808 nm excitation and NIR-I emission; II-A and III-A, respectively) or longer emission (670 nm excitation and NIR-II or NIR-II LP, I-B or I-C, respectively) reduces the autofluorescence signal in the abdomen by two orders of magnitude, even in mice fed normal chow. Switching from regular chow to a purified diet significantly lowered the autofluorescence of mice imaged under all but the III-B condition [Figs. 3(b) and 3(c)]; this difference was most practically impactful when illuminating or imaging using shorter wavelengths (i.e., I-A, II-A, or I-B). These results demonstrate that either changing diet or imaging in the NIR-II practically eliminates autofluorescence background at these short exposure times.

**Fig. 4:**
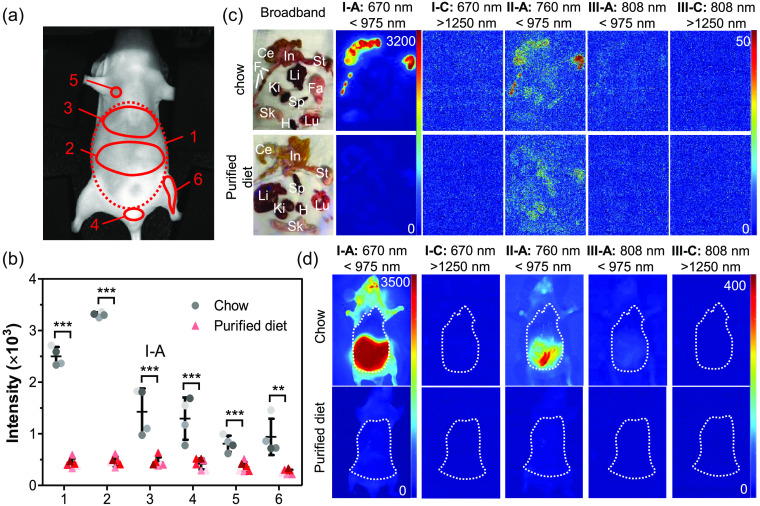
Impact of diet, excitation wavelength, and emission filter on region and organ autofluorescence. (a) Regions of interest (ROIs) indicated by numbered red circles. (b) Comparison of average ROI pixel intensity when imaging with 670 nm excitation and emission filter NIR-I (<975  nm, I-A). Exposure time: 0.01 s. Dots with different shaded colors represent autofluorescence intensity in different mice. n=4 (chow) and n=6 (purified). (c) Broadband and autofluorescence images of dissected organs from mice fed chow or purified diet acquired with selected excitation/emission filters. Li, liver; Lu, lung; H, heart; Sp, Spleen; Ki, kidney; St, stomach; In, intestine; Ce, cecum; Sk, skin; F, feces; and Fa, fat pad. Exposure time: 0.001 s. Note that the intensity scale for the I-A images is from 0 to 3200 counts and all other images are on a scale of 0 to 50 counts. (d) Autofluorescence images of mice fed chow (top row) or purified diet (bottom row) during dissection. Skin was removed from abdomen while leaving the peritoneal wall intact prior to imaging. The dotted lines indicate the region of exposed (skin-free) abdomen.

Next, different ROIs were selected to examine the spatial distribution of autofluorescence (Fig. 4). Six ROIs were drawn to represent the autofluorescence around the whole abdomen (ROI-1), digestive tract (central abdomen, ROI-2), liver (upper abdomen, ROI-3), genital region (ROI-4), skin (neck, ROI-5), and hindlimb (ROI-6). Consistent with the results from the whole abdomen (ROI-1), the average autofluorescence intensity of different ROIs was highest when imaging under the I-A conditions in mice with the chow or purified diet. Among different ROIs, the region corresponding to the digestive tract (ROI-2) autofluoresced brightly in mice with the chow diet, whereas the autofluorescence of other ROIs remained comparable to each other; little to no difference in the autofluorescence intensity was observed between ROIs in mice with the purified diet (data not shown). Comparison of the autofluorescence in the I-A condition showed that there were significant differences in the intensity between mice with the chow and purified diets in each of the ROIs [Fig. 4(b)]. Imaging of the organs *ex vivo* confirms that the bright autofluorescence is isolated to the digestive organs of chow-fed mice, including the stomach and intestines; feces visibly autofluoresces as well [Fig. 4(c)]. Quantitative comparison of these images confirms that most autofluorescence in chow-fed mice is eliminated with a switch to a purified diet (data not shown). The higher autofluorescence in chow-fed mice even when looking at regions away from the digestive tract arises from larger skin autofluorescence when mice are fed a chow-based diet.^18^^,^^22^ Thus unlike the significant ROI autofluorescence differences in live mice, the average autofluorescence intensity of most dissected organs other than intestine, stomach, and skin displayed only slight differences between mice in the two groups (data not shown). Chow-fed mice imaged during dissection with the skin over the abdomen removed, and the peritoneum and organ positions intact, exhibited much lower autofluorescence where the skin had been removed, visually demonstrating the impact of skin autofluorescence [Fig. 4(d)].

**Fig. 5:**
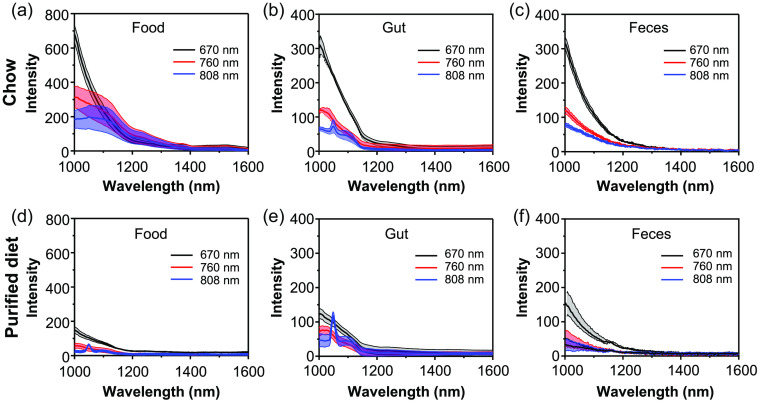
Spectra of food-based autofluorescence. Autofluorescence spectra of the food, intestines, and feces of (a)–(c) mice fed chow or (d)–(f) purified diet, respectively, from 1000 to 1600 nm following laser excitation with 670, 760, or 808 nm light. Food and feces was imaged with a 0.5 s exposure time, and gut autofluorescence [ROI-2 from Fig. 2(a)] was imaged with a 2 s exposure time. Spectra are mean ± std dev of n=3 independent samples/mice.

As seen in previous studies, imaging with longer excitation wavelength or NIR-II emission filters largely suppresses the autofluorescence signal.^11^^,^^24^ Hyperspectral analysis clearly shows the wavelength dependence of autofluorescence (Fig. 5). Almost no autofluorescence is observed in the NIR-II LP region (>1250  nm) regardless of the excitation wavelength. In contrast, measurable amounts of autofluorescence are observed from a chow pellet, the digestive tract of a chow-fed mouse, and the feces of a chow-fed mouse between 1000 and 1250 nm. As excitation wavelength redshifted from 670 to 760 nm and to 808 nm, significantly lower autofluorescence of bulk chow was observed <1100  nm, consequently resulting in autofluorescence decrease in mice intestine and feces.

## 4 Impact of Autofluorescence on Imaging with Contrast Agent

**Fig. 6:**
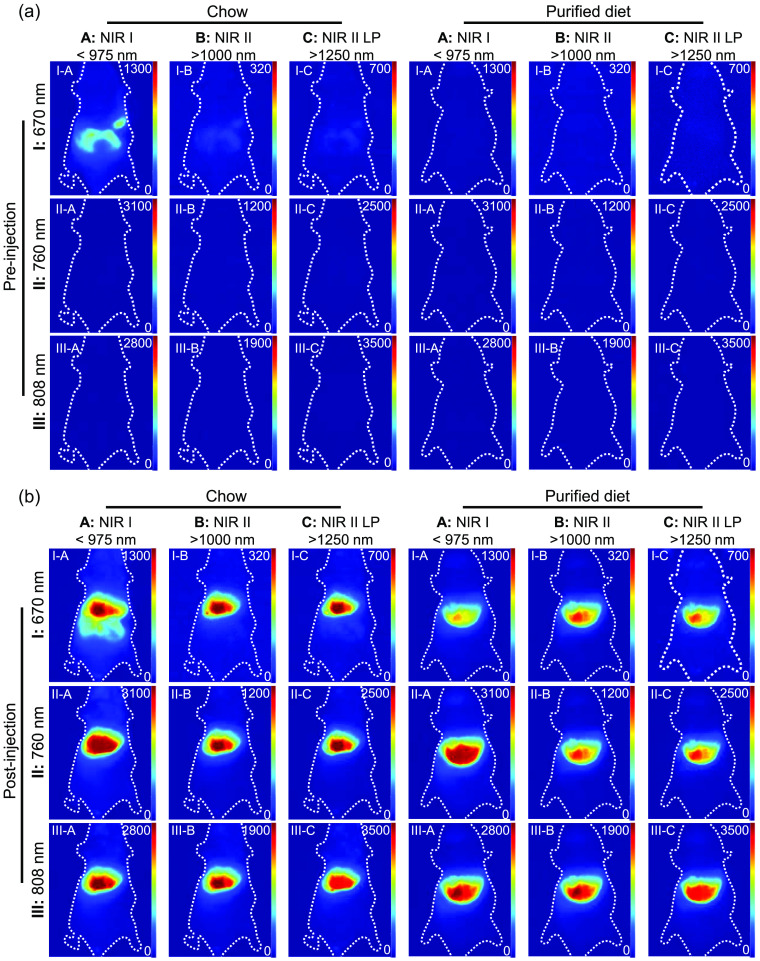
Comparison of ICG imaging in mice with the chow or purified diet. Mice are imaged with different imaging conditions (a) before and (b) 10 min after tail vein injection of ICG (100  μg/mL, 100  μL, 0.5  mg/kg). Exposure time: 0.0005 s for I-A, II-A, and III-A filters; 0.002 s for I-B, II-B, and III-B filters; 0.2 s for I-C, II-C, and III-C filters. The white dotted line indicates the position of the mice. Note that the pixel intensity scale is optimized for each imaging condition and is the same for the chow-fed and purified diet-fed mice in both the pre- and postinjection images.

ICG, a US Food and Drug Administration-approved fluorescence-imaging agent, has widely been used for clinical diagnosis, such as the evaluation of cardiac output and liver function.^30^^,^^31^ ICG, with its excitation peak at around 810 nm, has been widely used for NIR-I imaging^32^^,^^33^ and has recently been explored for NIR-II imaging, with studies demonstrating improved outcomes using only the long-wavelength emission tail.^27^^,^^28^ In this context, we used fluorescence imaging with ICG to examine the impact of background autofluorescence under different imaging conditions. We observed a substantial mismatch between the ICG distribution area and the intensity observed in the I-A condition (670 nm excitation and <975  nm emission) of mice fed chow, whereas no such obvious confounder is seen among images with other filters (Fig. 6). The autofluorescence imaging results discussed above and preinjection images indicate that the interference arises from chow-based autofluorescence in the digestive system. The substantial chow autofluorescence hinders our ability to distinguish the ICG signal from the autofluorescence background using the I-A imaging condition.

**Fig. 7:**
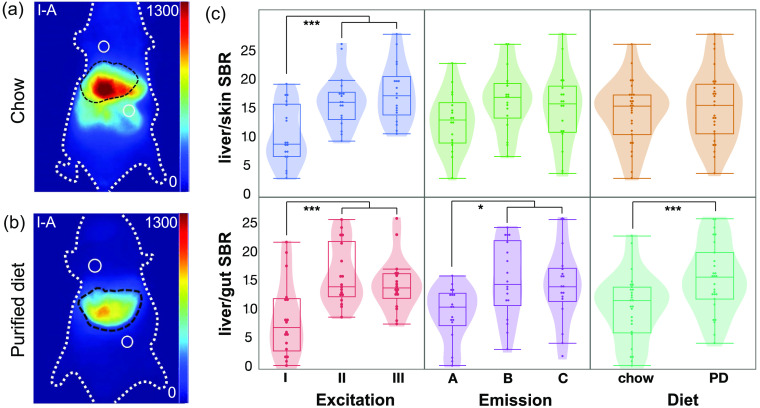
Signal-to-background quantification with ICG contrast. (a) Fluorescence images following ICG injection of mice fed chow and (b) purified diet (I-A; 670 nm excitation, NIR-I emission). Black dotted lines denote boundary of ICG signal; white solid circles represent ROIs for background signal evaluation. (c) Quantitative SBRs plotted against individual variables. Excitation wavelengths: 670 nm (I), 760 nm (II), and 808 nm (III). Acquisition filters: NIR-I (<975  nm, A), NIR-II (>1000  nm, B), and NIR-II LP (>1250  nm, C). PD = purified diet. n=3 mice on each diet, each imaged with all nine imaging conditions. *: p <0.05; ***: p<0.005.

The SBRs were calculated to illustrate the impact of background autofluorescence on image contrast. We chose two sites near the liver for the background signal evaluation: an abdominal region representative of gut autofluorescence and a region above the liver that represents the typical background autofluorescence intensity of other regions, primarily skin (Fig. 7). When comparing the ICG fluorescence to the autofluorescence of the skin [Fig. 7(c), top], the 670-nm excitation yielded significantly lower SBR than excitation at either 760 or 808 nm (p<0.005), but changing the emission window or diet alone did not show any significant difference in SBR. When comparing the ICG intensity in the liver to the background autofluorescence of the gut, excitation at 670 nm (p<0.005), NIR-I emission (p<0.05), and the chlorophyll-containing chow (p<0.005) all contributed to a lower SBR compared with the other experimental options [Fig. 7(c), bottom].

Due to the intense autofluorescence of the digestive tract in mice fed chow, the liver/gut SBR averaged 1.35 when imaging with I-A conditions (670 nm excitation and <975  nm emission), far from the Rose criterion (SBR of 5) to distinguish image features with 100% certainty.^34^^,^^35^ Mice fed a purified diet exhibited little difference between the liver/gut and liver/skin SBRs, consistent with a low variation in the background autofluorescence, as seen in Fig. 4(b). In contrast, even when imaging with the NIR-II LP, the average background intensity was significantly higher with the chow diet at all three excitation wavelengths [Fig. 3(d)]. For short-exposure times, changing the diet may not be practically necessary, but as exposure time is increased, lowering the background signal through a diet change may still provide an SBR benefit in NIR-II imaging.

## 5 Conclusion

We evaluated the impact of diet, excitation wavelength, and emission wavelength on the autofluorescence of mice in the NIR-I and NIR-II biological imaging windows to optimize generally applicable NIR imaging conditions. Following illumination with power density-matched ($1\;\mathrm{mW}/{\mathrm{mm}}^{2}$) laser excitation at 670, 760, or 808 nm, we compared the autofluorescence in the NIR-I (<975  nm), NIR-II (>1000  nm), and NIR-II LP (>1250  nm) regions. Autofluorescence was quite intense, especially in the NIR-I, when excited with a 670-nm laser due to the autofluorescence of chlorophyll in regular chow. The impact of this background was ameliorated by changing the rodent diet to eliminate chlorophyll and/or shifting the excitation and/or emission to longer wavelengths. Autofluorescence was barely above the background signal of the detector when longer excitation wavelengths (760 or 808 nm) were paired with NIR-II imaging, regardless of diet. An example of imaging with the dye ICG demonstrates the improvements to SBR and ROI identification enabled by intentional choices to lower autofluorescence. This systematic comparison of imaging conditions and diet highlights the improvements to fluorescence imaging that can be gained through background mitigation.
